# Rare Case of a Very Large Angioleiomyoma of the Dorsum Foot

**DOI:** 10.7759/cureus.14525

**Published:** 2021-04-16

**Authors:** Kevin Ho, Mark Ireland, Paul S Armanasco

**Affiliations:** 1 Podiatric Medicine, The University of Western Australia, Perth, AUS; 2 Podiatric Surgery, Australasian College of Podiatric Surgeons, Perth, AUS; 3 Podiatry, Curtin University, Perth, AUS

**Keywords:** angioleiomyoma, soft tissue tumor, leiomyoma, benign, foot

## Abstract

Angioleiomyomas are relatively rare benign smooth muscle soft tissue tumors which often occur on the extremities. They are rarely diagnosed preoperatively as clinical and radiological examination is often nonspecific and inconclusive. An 80-year-old male presented with a 10-year history of a progressively growing and symptomatic lesion on his right dorsal foot within the first intermetatarsal space. The preoperative diagnosis was suspected to be a neurogenic schwannoma arising from the deep peroneal nerve. Simple excision and histopathology confirmed a diagnosis of angioleiomyoma with nil recurrence or complications. The size of the angioleiomyoma was the second largest reported in literature to date. Angioleiomyomas are often misdiagnosed, and a degree of suspicion should be maintained in patients presenting with lower extremity growing soft tissue tumors.

## Introduction

Angioleiomyomas, or vascular leiomyomas, are benign smooth muscle soft tissue tumors which arise from the tunica media of subcutaneous blood vessels [1-3]. They account for 4.4% of all benign soft tissue tumors and 0.2% of benign foot lesions, but are primarily found in the lower extremity [1,4]. Angioleiomyomas typically present as a firm, rounded mobile nodule which arise commonly in the deep layers of the dermis or subcutaneous tissues [5]. Though sometimes asymptomatic, they are slow-growing masses which can often result in pain, discomfort, footwear limitations, cosmesis concerns, and nerve entrapment [1-3,6]. The majority of angioleiomyomas are small, ranging only from 0.5 to 2 cm in diameter [3,5].

Due to their rare occurrence, lack of awareness from clinicians, and paucity of published literature, angioleiomyomas are rarely diagnosed preoperatively. This highlights the importance of better characterizing the diagnosis and treatment of angioleiomyomas in the clinical setting to expand the awareness of this pathology as a differential diagnosis for lower extremity soft tissue tumors. An accurate diagnosis prevents delay in treatment, improves patient outcomes, and excludes malignant involvement.

The current literature of angioleiomyomas is predominantly limited to single case studies, with variance in reported clinical presentations and imaging findings. To enhance the general awareness of angioleiomyomas in clinical practice, further studies illustrating its unique presentation will improve our understanding of its pathogenesis, natural history, and treatment outcomes. This article details a case report of an exceptionally large, long-standing, and symptomatic angioleiomyoma of 4.6 cm diameter on the dorsal foot. A pedal angioleiomyoma of this size is second only to the reported case in 1994 by Habershaw et al. which measured 6 cm in diameter [7].

## Case presentation

**Figure 1:**
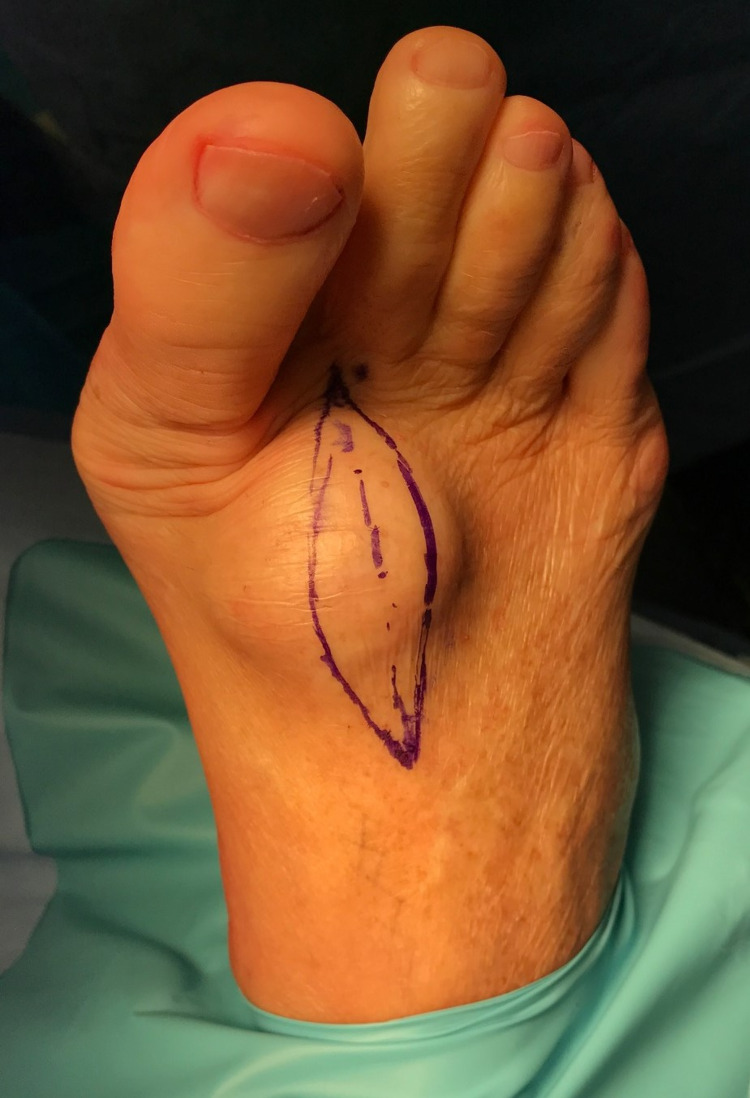
Soft tissue mass on the patient’s right dorsum foot within the first intermetatarsal space.

An 80-year-old Caucasian male presented with a 10-year history of a progressively growing and symptomatic lesion on the dorsum of his right foot within the first intermetatarsal space (Figure 1). The patient was relatively healthy without major comorbidities, including no personal or familial history of prior deep vein thrombosis, smoking, recent surgery, blood clotting disorders, diabetes, or respiratory conditions. He was classified with an American Society of Anaesthesiologist (ASA) category II, having mild systemic disease.

The patient reported ongoing irritation and limitations in footwear, with concurrent paraesthesia in the first web space. He was concerned about the progressive growth of the lesion within recent years. He recalled nil previous trauma or foot and ankle surgery. On examination, the large soft tissue tumor was dome-shaped, immobile, and solid. Direct palpation elicited mild pain and recreated his paraesthesia in the first web space which identified involvement of the deep peroneal nerve.

Ultrasound imaging demonstrated a clearly demarcated asymmetrical dumbbell-shaped mass with heterogenous echogenicity. There was definite internal vascularity and no cystic component. The mass did not appear to arise from the underlying joints. Ultrasound imaging was inconclusive, and the sonographer recommended further magnetic resonance imaging (MRI) and fine needle aspiration (FNA) biopsy.

**Figure 2:**
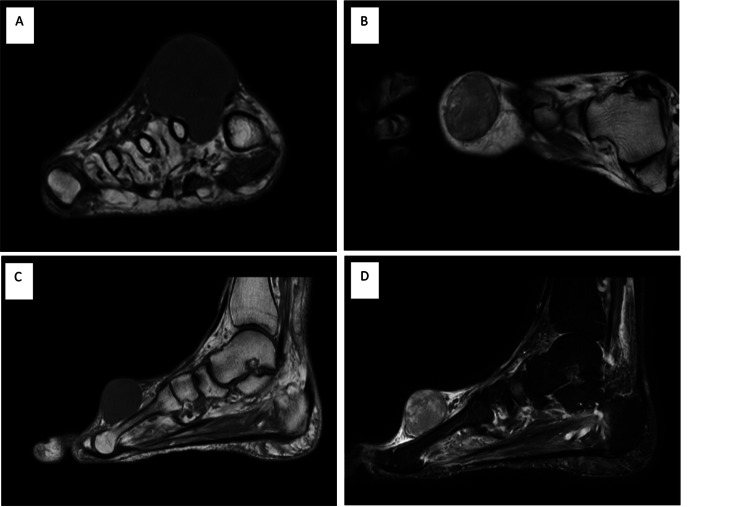
MRI of the dorsum soft tissue tumor. Coronal T1-weighted (A), transverse T1-weighted (B), and sagittal T1-weighted (C) images demonstrate the hypointense encapsulated mass. The sagittal T2-weighted image (D) demonstrates mixed intensity. MRI, magnetic resonance imaging

MRI identified a nonspecific large dumbbell-shaped solid soft tissue tumor measuring 4 × 4 × 4 cm within the first intermetatarsal space (Figure 2). The mass was well-encapsulated T1 hypointense, T2 mixed intensity, short-TI inversion recovery hyperintense and demonstrated extensive enhancement after administration of gadolinium.

FNA biopsy with histopathological testing was performed which confirmed a nonspecific benign spindle cell lesion with fibromyxoid stroma. Both MRI and FNA biopsy testing were inconclusive, suggestive of differential diagnoses of giant cell tumor, desmoid tumor, tophi, or a neuroma. Clinical and investigative testing suggested a provisional preoperative diagnosis of a deep peroneal nerve schwannoma.

**Figure 3:**
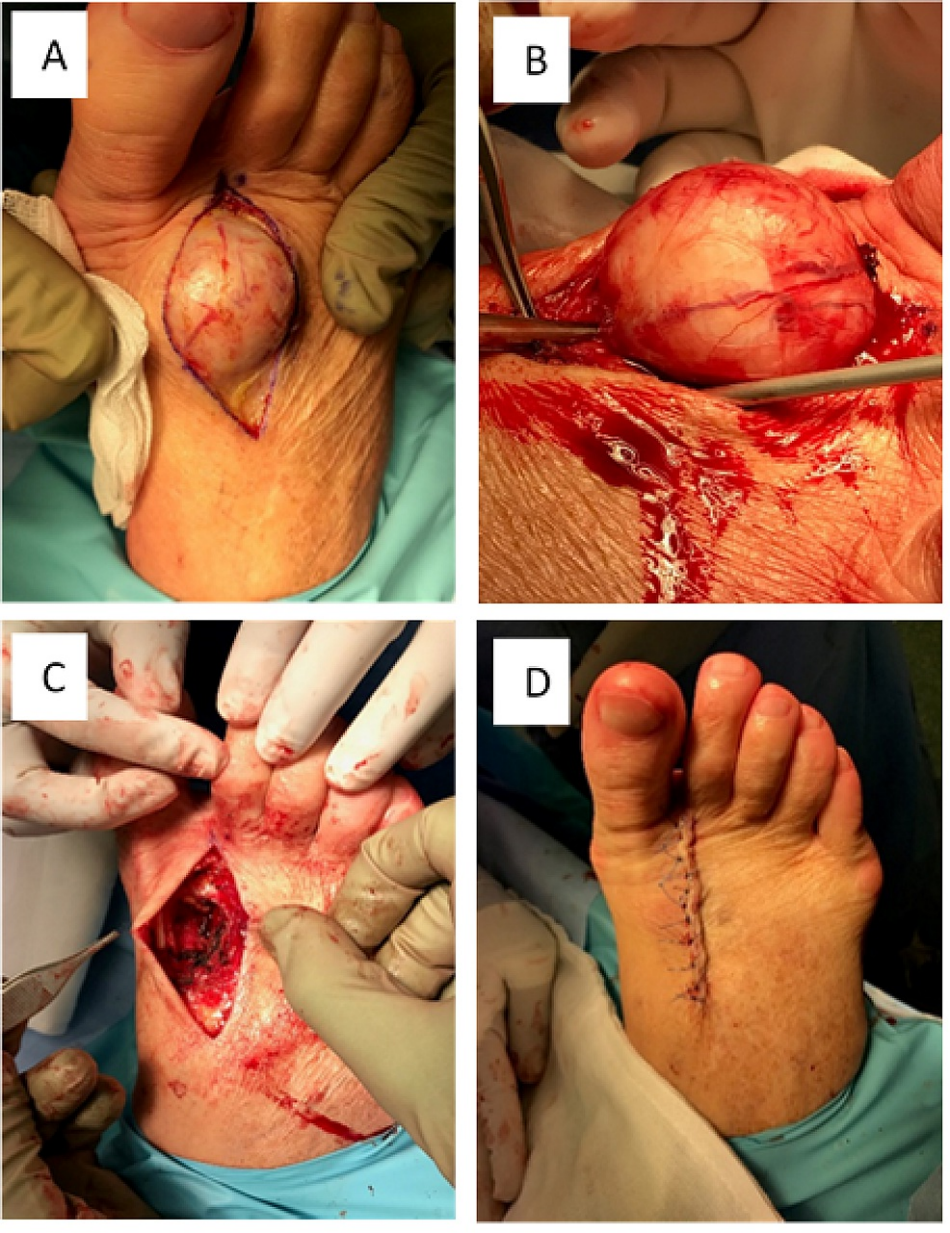
Surgical approach for excision of the mass. Intraoperative visualisation of the well-encapsulated large benign tumor.

The large mass was excised under general anesthesia with a local anesthetic field block (8 mL of 0.5% bupivacaine with 1:200000 adrenaline) without complication. The procedure was approached with a dorsal ellipse positioned at the lateral border of the mass (Figure 3A). The well-encapsulated and highly vascular solid mass was exposed following blunt dissection, mobilized, and sharply excised en bloc (Figure 3B). Meticulous hemostasis was ensured to prevent hematoma formation (Figure 3C). The wound was flushed and closed with 4/0 vicryl and 4/0 prolene sutures (Figure 3D).

**Figure 4:**
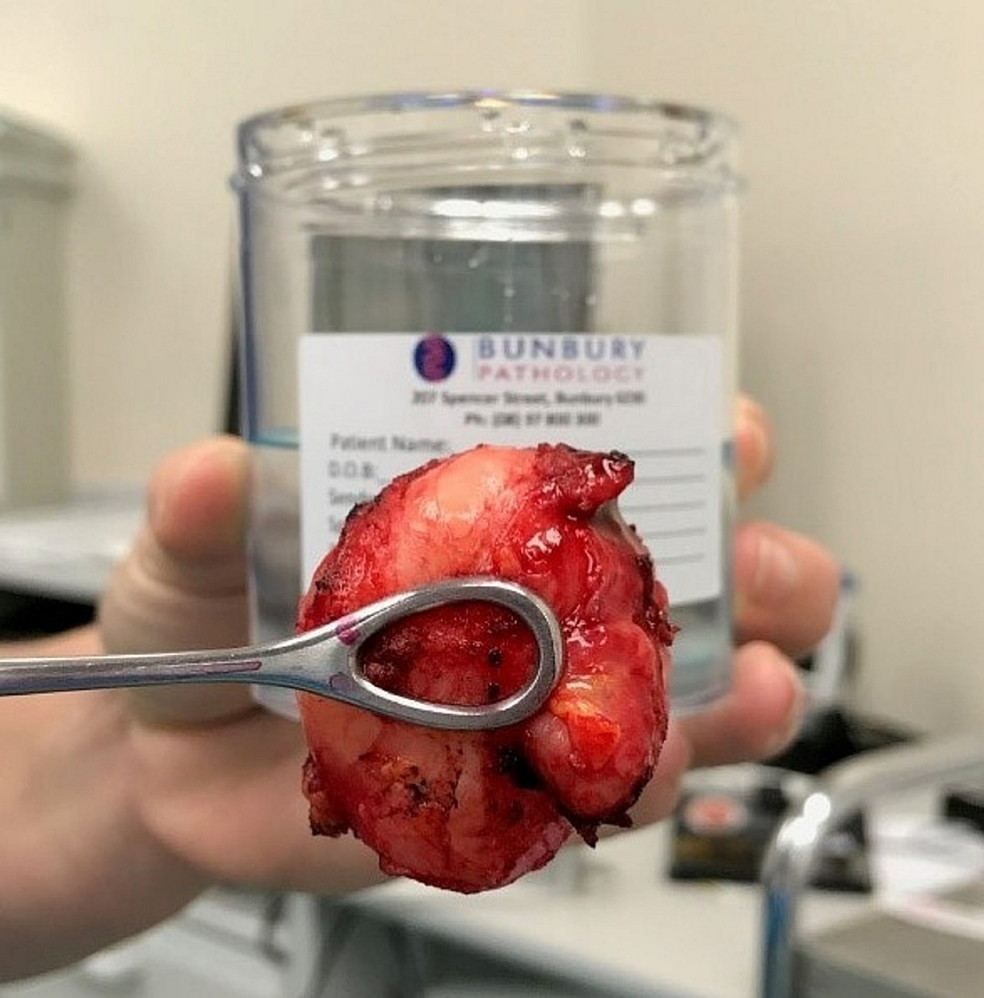
Mass excised and sent for histopathology. The mass was later identified as a benign angioleiomyoma.

The large mass (46 × 36 × 30 mm) was sent for histopathology, where the sections demonstrated a circumscribed bland spindle cell proliferation with low cellularity (Figure 4). The proliferating cells were composed of spindle cells in bundles and fascicles, elongated nuclei which had rounded ends, and fibrillar stroma. The section was admixed with ectatic blood vessels which were lined by regular endothelial cells. The histopathology findings were consistent with a postoperative diagnosis of a benign angioleiomyoma. Postoperative healing was uncomplicated and there were nil symptoms or evidence of regrowth at 12 months review.

## Discussion

The etiology of angioleiomyomas remains indefinite, with literature theorizing the involvement of vascular malformations, trauma, venous stasis, infection, and hormonal imbalances in females [2,8,9]. An autosomal dominant inheritance genetic predisposition has also been cited, though further current literature is warranted [10,11]. To further support the possible genetic component, Sweeney and Keating reported angioleiomyomas to be 10 times more prevalent in East African populations when compared to Caucasians [12]. The specific etiology of the case angioleiomyoma is difficult to isolate, considering its chronicity and lack of reported familial history. Irrespective of the case presentation, angioleiomyomas typically occur in middle-aged females [2,8,9].

Currently, the literature regarding MRI features of angioleiomyomas continue to be scarce, and thus distinguishing between various soft tissue tumors can be challenging [13]. Similar to the case findings, current literature reports common MRI findings to include a well-demarcated mass of isointense or hypointense signal on T1-weighted images, heterogeneous high signal on T2-weighted images, and adjacent tortuous vascular structures, often localized to the extremities [13]. However, MRI findings are often varied case-by-case and nonspecific, potentially masquerading as giant cell or neurogenic tumors [13]. Thus, although MRI visualization can aid in surgical planning, findings are often inconclusive.

Literature consensus reports histopathological testing to be the gold standard in definitively diagnosing angioleiomyomas [4,14,15]. Demonstrated by the case findings, literature reports angioleiomyomas to consistently demonstrate spindle-shaped cells set in bundles with elongated cigar-shaped nuclei and surrounding vascular channels lined by regular endothelial cells [4,14,15]. Other reported histological findings include myxomatous or hyaline degeneration [4,14,15]. Current literature reports simple excision and primary closure to be the treatment of choice, with rare recurrence rates (approximately 0.36%) and minimal morbidity [3]. Malignant recurrence or transformation are uncommon and account for 7% of all soft tissue sarcomas [16].

This article highlights the diagnostic challenges of angioleiomyomas due to their relative rarity, paucity of radiological literature and nonspecific nature of clinical and diagnostic examinations. Despite preoperative clinical examination, radiological tests, and biopsies, the case study diagnosis of an angioleiomyoma was unsuspected. Angioleiomyomas should be considered as a potential differential when examining slow-growing soft tissue tumors of the lower extremity. A high index of suspicion is warranted to exclude malignant or aggressive tumors. Further research is justified to aid clinicians in obtaining an accurate preoperative diagnosis to ensure a suitable treatment plan.

## Conclusions

Angioleiomyomas are relatively rare benign soft tissue tumors which can occur in the extremities of middle-aged people. Preoperative diagnosis remains a diagnostic challenge, with clinical and radiological examination being ambiguous and nonspecific. Histopathology is the gold standard in obtaining a definitive diagnosis, and simple excision is highly curative. This article reports a case of the second largest angioleiomyoma of the dorsum foot where simple excision was successful with nil recurrence or complications. Angioleiomyomas are often misdiagnosed, and a degree of suspicion should be maintained in patients presenting with lower extremity growing soft tissue tumors.

## References

[1] Moriarty J, Sottile J, Thakurdial T, Wrzolek M, Liu Y (2019). Angioleiomyoma of the foot. J Am Podiatr Med Assoc.

[2] Yates BJ (2001). Angioleiomyoma: clinical presentation and surgical management. Foot Ankle Int.

[3] Baarini O, Gilheany M (2016). Angioleiomyoma of the plantar-medial arch: a case report. J Clin Diagn Res.

[4] Santucci A, Albini M, Ventura A, De Palma L (2000). Clinical and histological features of vascular leiomyoma of the foot: case report and literature review. Foot Ankle Surg.

[5] Gajanthodi S, Rai R, Chaudhry RK (2013). Vascular leiomyoma of foot. J Clin Diagn Res.

[6] Macdonald DJ, Holt G, Vass K, Marsh A, Kumar CS (2007). The differential diagnosis of foot lumps: 101 cases treated surgically in North Glasgow over 4 years. Ann R Coll Surg Engl.

[7] Habershaw G, Hurchik J, Nasser I (1994). Pedal leiomyoma. J Foot Ankle Surg.

[8] Ramesh P, Annapureddy SR, Khan F, Sutaria PD (2004). Angioleiomyoma: a clinical, pathological and radiological review. Int J Clin Pract.

[9] Lepoff A, Makarov V, Williams M (2018). Angioleiomyoma of the plantar foot. J Am Podiatr Med Assoc.

[10] Fox SB, Heryet A, Khong TY (1990). Angioleiomyomas: an immunohistological study. Histopathology.

[11] Hachisuga T, Hashimoto H, Enjoji M (1984). Angioleiomyoma. A clinicopathologic reappraisal of 562 cases. Cancer.

[12] Sweeney J, Keating SE (1983). Angioleiomyoma. J Foot Surg.

[13] Yoo HJ, Choi JA, Chung JH (2009). Angioleiomyoma in soft tissue of extremities: MRI findings. AJR Am J Roentgenol.

[14] Ramesh P, Annapureddy SR, Khan F, Sutaria PD (2004). Angioleiomyoma: a clinical, pathological and radiological review. Int J Clin Pract.

[15] Kang BS, Shim HS, Kim JH (2019). Angioleiomyoma of the extremities: findings on ultrasonography and magnetic resonance imaging. J Ultrasound Med.

[16] Woo KS, Kim SH, Kim HS, Cho PD (2014). Clinical experience with treatment of angioleiomyoma. Arch Plast Surg.

